# Regulation of AMPK activation by extracellular matrix stiffness in pancreatic cancer

**DOI:** 10.1016/j.gendis.2023.05.022

**Published:** 2023-07-14

**Authors:** Xin Xu, Yuan Fang, Somaira Nowsheen, Ye-Xiong Li, Zhenkun Lou, Min Deng

**Affiliations:** a State Key Laboratory of Molecular Oncology and Department of Radiation Oncology, National Cancer Center/National Clinical Research Center for Cancer/Cancer Hospital, Chinese Academy of Medical Sciences and Peking Union Medical College, Beijing 100021, China; b Department of General Surgery, Shanghai General Hospital, Shanghai Jiaotong University School of Medicine, Shanghai 200080, China; c Department of General Surgery, Zhongshan Hospital, Fudan University, Shanghai 200032, China; d Department of Dermatology, University of California San Diego, San Diego, CA 92093, USA; e Department of Oncology, Mayo Clinic, Rochester, MN 55905, USA

**Keywords:** AMPK activity, Cellular metabolic switch, ECM stiffness, Hippo kinase signaling, Pancreatic ductal adenocarcinoma

## Abstract

The adenosine monophosphate (AMP)-activated protein kinase (AMPK) sits at a central node in the regulation of energy metabolism and tumor progression. AMPK is best known to sense high cellular ADP or AMP levels, which indicate the depletion of energy stores. Previous studies have shown that the low expression of phosphorylated AMPK is associated with a poor prognosis of pancreatic cancer. In this study, we report that AMPK is also highly sensitive to extracellular matrix (ECM) stiffness. We found that AMPK is activated in cells when cultured under low ECM stiffness conditions and is functionally required for the metabolic switch induced by ECM stiffness. This regulation of AMPK requires the Hippo kinases but not LKB1/CaMKKβ. Hippo kinases directly phosphorylate AMPKα at Thr172 to activate AMPK at low ECM stiffness. Furthermore, we found AMPK activity is inhibited in patients with pancreatic ductal adenocarcinoma (PDAC) with high ECM stiffness and is associated with a poor survival outcome. The activation of Hippo kinases by ROCK inhibitor Y-27632 in combination with the mitochondrial inhibitor metformin synergistically activates AMPK and dramatically inhibits PDAC growth. Together, these findings establish a novel model for AMPK regulation by the mechanical properties of ECMs and provide a rationale for simultaneously targeting the ECM stiffness–Hippo kinases–AMPK signaling and low glucose–LKB1–AMPK signaling pathways as an effective therapeutic strategy against PDAC.

## Introduction

AMPK is a key regulator of cellular metabolism, and its deregulation is linked to metabolic diseases such as obesity, diabetes, inflammation, and cancer.^1^, ^2^, ^3^ As a protein kinase complex, AMPK activates catabolic processes and inhibits anabolic processes to balance cellular energy, impacting gene transcription, signaling transduction, and cellular fate. It also functions as a metabolic tumor suppressor, triggering cellular metabolic checkpoints that affect cell growth, cell death, and autophagy through its actions on modulators such as mTORC1, AKT, p53, and ULK1.^4^

AMPK is composed of catalytic α, regulatory β, and nucleotide-binding γ subunits.^5^^,^^6^ It senses changes in the cellular levels of AMP, ADP, and ATP through a multi-step process.^7^ First, the binding of AMP or ADP to the γ-subunit promotes Thr172 phosphorylation in the α-subunit by the upstream kinase LKB1, activating AMPK activity by up to 100-fold *in vitro*.^8^, ^9^, ^10^, ^11^, ^12^ Second, the binding of AMP or ADP to the γ-subunit causes a conformational change that protects Thr172 from dephosphorylation by protein phosphatases.^13^^,^^14^ Lastly, binding of AMP leads to allosteric activation of AMPK by up to 10-fold.^10^ In addition to the canonical pathways above, AMPK also senses other cellular signals, such as Ca^2+^/calmodulin-dependent protein kinase β (CaMKK β) and fructose-1,6-bisphosphate (FBP).^3^^,^^15^, ^16^, ^17^, ^18^ In the process of maintaining energy homeostasis, both the Hippo kinase pathway and AMPK are activated by a cellular energy starvation signal, resulting in yes-associated protein (YAP) phosphorylation and inactivation.^19^^,^^20^ Recent research confirmed that AMPK is involved in the glycolysis pathway in pancreatic cancer.^21^ In addition, AMPK has been identified as playing an important role in the regulation of autophagy,^22^^,^^23^ ferroptosis sensitivity,^24^ and the activation of innate immune signal TANK-binding kinase 1 (TBK1)^25^ in pancreatic cancer. The regulation of AMPK by biochemical signaling is well-studied, but its regulation by mechanical cues in tumor microenvironments is not well understood. Much attention has been focused on the link between mitochondrial mechanotransduction and AMPK^26^, ^27^, ^28^ as well as the necessity of E-cadherin/LKB1 signaling.^26^^,^^29^ In this study, we demonstrate that extracellular matrix (ECM) stiffness regulates AMPK activity through the ECM stiffness–Hippo kinases signaling pathway. Our data suggest that simultaneously targeting the ECM stiffness-Hippo kinases-AMPK signaling and the low glucose-LKB1-AMPK signaling can be an effective therapeutic strategy against tumors with high stiffness, such as pancreatic ductal adenocarcinoma (PDAC).

## Material and methods

### Materials

The following antibodies were used: total AMPKα (#5831; Cell Signaling Technologies, MA, USA), AMPKα1 (#2795; Cell Signaling Technologies), AMPKα2 (#2757; Cell Signaling Technologies), Phospho-AMPKα (Thr172) (40H9) Rabbit mAb (#2535; Cell Signaling Technologies), Phospho-Acetyl-CoA Carboxylase (Ser79) antibody (#3661; Cell Signaling Technologies), ACC1 (#4190; Cell Signaling Technologies), actin (A2228; Sigma–Aldrich, MO, USA), CaMKKβ (sc-100364; Santa Cruz Biotechnology, TX, USA), FLAG (F1804; Sigma–Aldrich), HA (#5017; Cell Signaling Technologies), and Myc (9E10; Santa Cruz Biotechnology, TX, USA). The HRP-conjugated second antibodies (goat anti-mouse IgG, Cat. 115-035-003, 1:5000 for immunoblotting; goat anti-rabbit IgG, Cat. 111-035-003, 1:5000 for IB) were procured from Jackson ImmunoResearch (PA, USA).

Cell-permeable C3 transferase (Cat. # CT04) was purchased from Cytoskeleton Inc (CO, USA). Actin polymerization inhibitor Latrunculin B (Lat B; 10,010,631) was purchased from Cayman Chemical (MI, USA). ROCK inhibitor Y-27632 (S1049) and Rac1-GEF inhibitor NSC 23766 (S8031) were purchased from Selleckchem (TX, USA); calcium ionophore ionomycin (I3909) and microtubule polymerization inhibitor Nocodazole (M1404) were purchased from Sigma–Aldrich; and SAMS peptide (ab120182) was purchased from Abcam (MA, USA). Purified protein kinases (MST1, MST2, MAP4K1, MAP4K2, MAP4K3, MAP4K4, MAP4K6, and MAP4K7) were all obtained from SignalChem Biotech Inc (Canada). Finally, AMPK (α1/β1/γ1) complex (14–840) was purchased from Millipore Sigma (MA, USA).

### AMPK activity assay

To measure AMPK activity in cell lysates (100 μg), the AMPK kinase complex was immunoprecipitated using anti-AMPKα1 and anti-AMPKα2 antibodies overnight. Protein A/G beads were then added and mixed for 2 h. The beads were rinsed with cold NETN lysis buffer (100 mM NaCl, 20 mM Tris-Cl, pH 8.0, 0.5 mM EDTA, 0.5% (v/v) Nonidet P-40) five times and then balanced with *in vitro* kinase reaction buffer an additional two times. The AMPK kinase activity was determined by measuring the intensity of SAMS phosphorylation using the following reaction mix: 15 mM HEPES (pH 7.0), 18.75 mM MgCl_2_, 1 mM dithiothreitol, 125 μM cold ATP, 12.5 μCi of radiolabeled ATP (hot), and 150 μM AMP. Aliquots (10 μL) were harvested at indicated time points to calculate the ^32^P incorporation rate.

To evaluate the effect of Hippo kinase on AMPK activation, 0.2 μg purified AMPK (α1/β1/γ1) complex was incubated with a specified amount of Hippo kinases in kinase buffer (15 mM HEPES (pH 7.0), 5 mM MnCl_2_, 5 mM MgCl_2_, 1 mM ATP, and 1 mM dithiothreitol) at 37 °C for 2 h. At the end of the reaction, 0.05 μg AMPK was aliquoted and AMPK activity was calculated using the method described above.

### *In vitro* kinase screening assay

Panels of protein kinases (CMGC-1, V6854; CMGC-2, V6856; CAMK-1, V6932; CAMK-2, V6924; STE-1, V6916; TKL-1, V6914; AGC-1, V6858; AGC-2, V6910; Other/CK-1, V6918; and Other-2, V6926) were obtained from Promega Inc (WI, USA). First, 2 μL of kinases were incubated with 1 ug of GST-AMPKα1 protein in the kinase reaction buffer (15 mM HEPES (pH 7.0), 10 mM MnCl_2_, 10 mM MgCl_2_, 1 mM ATP, and 1 mM dithiothreitol) at 30 °C for 30 min. For PAK1/CDC42, 333.3 μM GTP was included in the kinase assay buffer. For DNA-PK, 33 μM calf thymus DNA was included in the kinase assay buffer; for CAMK2α, CAMK2γ, CAMK4, and DAPK1, 1.66 mM CaCl_2_ and 0.1 μg/μL calmodulin were added to the kinase assay buffer. The reactions were stopped by adding 2 × reducing sample buffer. The reaction products were separated via SDS-PAGE and blotted with anti-AMPKα pT172 antibody.

### *In vitro* kinase assay

100 ng purchased Hippo kinases were mixed with 1 μg of GST-AMPKα1 protein in the kinase assay buffer (15 mM HEPES (pH 7.0), 10 mM MnCl_2_,10 mM MgCl_2_, 1 mM ATP, and 1 mM dithiothreitol) at 30 °C for 30 min. The reactions were stopped by adding 2 × reducing sample buffer. The reaction products were boiled and separated via SDS-PAGE and immunoblotted with anti-AMPKα pT172 antibody.

### Cell culture and treatment

HEK293, HeLa, and A549 cell lines were purchased from ATCC (VA, USA) and cultured in DMEM and RPMI 1640 with 10% FBS. Control and MM-8KO cells (MST1/2 kinases and MAP4K knockout (KO) cell lines), RAP2 KO cells (RAP2 KO cell line), and YAP/TAZ KO cells (YAP/TAZ KO cell line) were kindly provided by Dr. Kunliang Guan (University of California, San Diego, CA, USA). AMPKα1/2 double KO mouse embryonic fibroblasts (MEFs) were kindly shared by Dr. Eduardo N. Chini (Mayo Clinic, MN). The cell lines were maintained in DMEM with 10% FBS.

For drug treatments, 10,000 cells per well were plated onto 6-well plates pretreated with 20 μg/mL bovine fibronectin (Sigma–Aldrich) at 25 °C for 1 h. Unless specified otherwise, drug concentrations and treatment conditions are indicated in the figure legend.

### Plasmids

FlAG-pLJM1-RAP2A, PJ3H-Mst1, pJ3M-Mst1 K59R, pJ3M-Mst2, pJ3H-Mst2 K56R, and pBabe_puro_DEST_Flag_TNIK were purchased from Addgene (MA, USA; # 19311, 12203, 12204, 12205, 12206, and 45276, respectively). FLAG-MAP4K1, FLAG-MAP4K2, FLAG-MAP4K3, FLAG-MAP4K4, and FLAG-MAP4K6 were all kindly shared by Dr. Kunliang Guan (University of California).

### shRNA-mediated knockdown of CaMKKβ

For CaMKKβ shRNA-mediated knockdown, pLKO.1-puro-shRNA lentivirus (#1: CCGGGTGAAGACCATGATACGTAAACTCGAGTTTACGTATCATGGTCTTCACTTTTT; #2: CCGGCGACCCTTTCTACTATGCATTCTCGAGAATGCATAGTAGAAAGGGTCGTTTTT) were packaged in 293T cells. After 48 h, the culture supernatant was harvested and incubated with A549 parental cells in the presence of 8 μg/mL polybrene to enhance infection efficiency. The infected cells were then selected with a medium containing 10 μg/mL puromycin for 7 d. CaMKKβ knockdown efficiency was determined via immunoblotting with an anti-CaMKKβ antibody.

### Cell culture with polyacrylamide-based hydrogels

Hydrogels depicting high (30 kPa) or low (1 kPa) ECM stiffness conditions were made using a previously described method^30^ by coating 10 μg/mL of human placenta fibronectin onto the sulfo-SANPAH-activated hydrogels.

### Protein synthesis with metabolic radiolabeling

To measure the rate of protein synthesis, cells were first cultured for 30 min in DMEM without methionine and labeled with radioactive ^35^S-Met for 20–30 min. The cells were then lysed in RIPA buffer. Cell lysates were centrifuged at 13,000 *g* for 10 min, and supernatants were harvested and spotted on a Whatman™ 3 MM paper. The paper was then placed in a 10% cold trichloroacetic acid (TCA) solution for 20 min. The paper was then transferred to a 5% TCA boiling solution for 15 min. The paper fragments were then rinsed once more with 5% TCA and 95% ethanol. The paper was then air-dried. Scintillation counting was used to measure the radioactivity of each sample, and the relative protein synthesis rate was normalized to the control group.

### Cell growth measurement

AMPKα^+/+^ and AMPKα^−/−^ MEF cells or MM0 and MM-8KO HEK293 cells were seeded on stiff (30 kPa) or soft (1 kPa) matrices. Live cells were counted and recorded as the mean ± SD at indicated time points.

### Immunoblotting and immunoprecipitation

In brief, cells were washed with cold PBS once and lysed with cold NETN buffer (pH 8.0; 100 mM NaCl, 20 mM Tris–HCl pH 8.0, 0.5% Nonidet P-40, 1 mM EDTA, 10 mM NaF, 50 mM β-glycerophosphate, and 1 mg/mL aprotinin). Cell lysates were centrifuged at 13,000 g for 30 min. Supernatants were harvested and then mixed with 2 μg of the indicated antibody at 4 °C overnight. Protein A/G sepharose beads were added and mixed for an additional 2 h. The protein A/G sepharose beads were then rinsed three to five times with cold NETN buffer, boiled for 5 min, and separated via SDS–PAGE. Standard immunoblotting was performed with the indicated antibodies.

### Glucose consumption rate, lactate production rate, and cellular ATP content measurement

Glucose consumption and lactate production rates were measured following the manufacturer's instructions (K606 and K607; Biovision Inc., CA, USA).^31^ Briefly, cells were cultured in DMEM with 10% FBS. After 48 h, the cell culture medium was harvested and centrifuged at 13,000 *g* for 30 min. Glucose and lactate concentrations in supernatants were analyzed using colorimetric kits. The level of ATP in cell extracts was determined using an ATP bioluminescence assay kit (ab113849; Abcam) following the protocol of the manufacturer.

### GST-AMPKα1/β1/γ1 protein purification

The pGEX-4T1-AMPK α1/β1/γ1 construct for bacterial expression was expressed in BL21 cells by inducing 150 μM IPTG (OD600–0.8) at 16 °C. Cells were lysed in GST-binding buffer (pH 8.0; 50 mM Tris-Cl, 1 mM PMSF, 0.1 mM EDTA, and 150 mM NaCl) followed by sonication for 10 min. Cell lysates were centrifuged at 45,000 *g* at 4 °C for 30 min, and the supernatant was collected and incubated with glutathione sepharose (GSH beads) at 4 °C for 8 h. GSH beads were subsequently rinsed five times with GST-binding buffer. The purified protein was then eluted with GST elution buffer (with 50 mM reduced glutathione). The proteins were dialyzed with PBS and frozen at −80 °C.

### Patients' specimens and follow-up

The study was approved by the Zhongshan Hospital Research Ethics Committee. Ninety PDAC patients were studied, all of whom underwent radical pancreatic cancer surgeries from September 2012 to May 2016 in the Department of Pancreatic Surgery at Zhongshan Hospital, Fudan University, China. All patients were followed up until February 1, 2019. Overall survival (OS) was calculated from the date of surgery to either the last follow-up or death. No preoperative anticancer treatment was administered, and all patients received postoperative gemcitabine chemotherapy (six cycles of 1000 mg/m^2^ intravenously over 30 min on days 1, 8, and 15 of a 28-day cycle). The 8th edition American Joint Committee on Cancer (AJCC) system was used to determine the clinicopathologic features.

### Tissue microarray immunohistochemistry staining

Tissue microarrays were created and stained using standard protocols. A high-sensitivity diaminobenzidine chromogenic substrate system was used for colorimetric visualization. The density of positive staining was measured using a computerized image system and captured using a Leica-CCD camera connected to a Leica-DM-IRE2 microscope (Leica, GER). Under high-power magnification, photographs of representative fields were captured using the Leica Q Win Plus software (version 3). The IHC staining results were evaluated by two independent, experienced pathologists. Finally, according to pAMPK and a-SMA staining, their low and high expression groups were set for comparisons.

### *In vivo* mice study using hydrogel

PANC1 cells (5 × 10^6^) were suspended in 50 μL PBS and embedded into 200 μL of hydrogels with different stiffness. After brief gelation (5 min), the hydrogel–cell mix was subcutaneously injected into nude mice. Control mice were treated with 200 mg/kg metformin in drinking water, 30 mg/kg Y27632 (every 3 d), or a combination of both as indicated for 4 wk. Tumor volume was measured at regular intervals using a Vernier caliper and calculated as length × width^2^/2. After 4 wk, the tumors were harvested and weighed.

### Statistical analyses

Statistical analyses were used to support the conclusions of this study. Unless otherwise specified, all experiments were performed at least three times. The sample size for each experiment is provided in the relevant figure legends and/or earlier and, unless otherwise specified, represents biological replicates or independent experiments performed on different days, each with technical triplicates. SPSS 20.0 for Windows (version 26.0; SPSS Inc) was used for the survival analysis of patients. Pearson *χ*^2^ or Fisher exact test was used to compare qualitative variables, and quantitative variables were analyzed by using *t*-test or Pearson correlation test. Kaplan–Meier analysis was used to determine the survival. Log-rank test was used to compare the survival of patients between subgroups, and Cox regression was used to perform multivariate analysis. All values are reported as mean ± SD. Statistical significance for all pairwise comparisons was evaluated with a two-tailed Student's *t*-test or two-way ANOVA test, and a *P* value < 0.05 was considered significant. To our knowledge, all the biochemical measurements provided data with a normal distribution and similar variance among the groups.

## Results

### AMPK is activated by low ECM stiffness and mediates cellular metabolic switch

**Figure 1:**
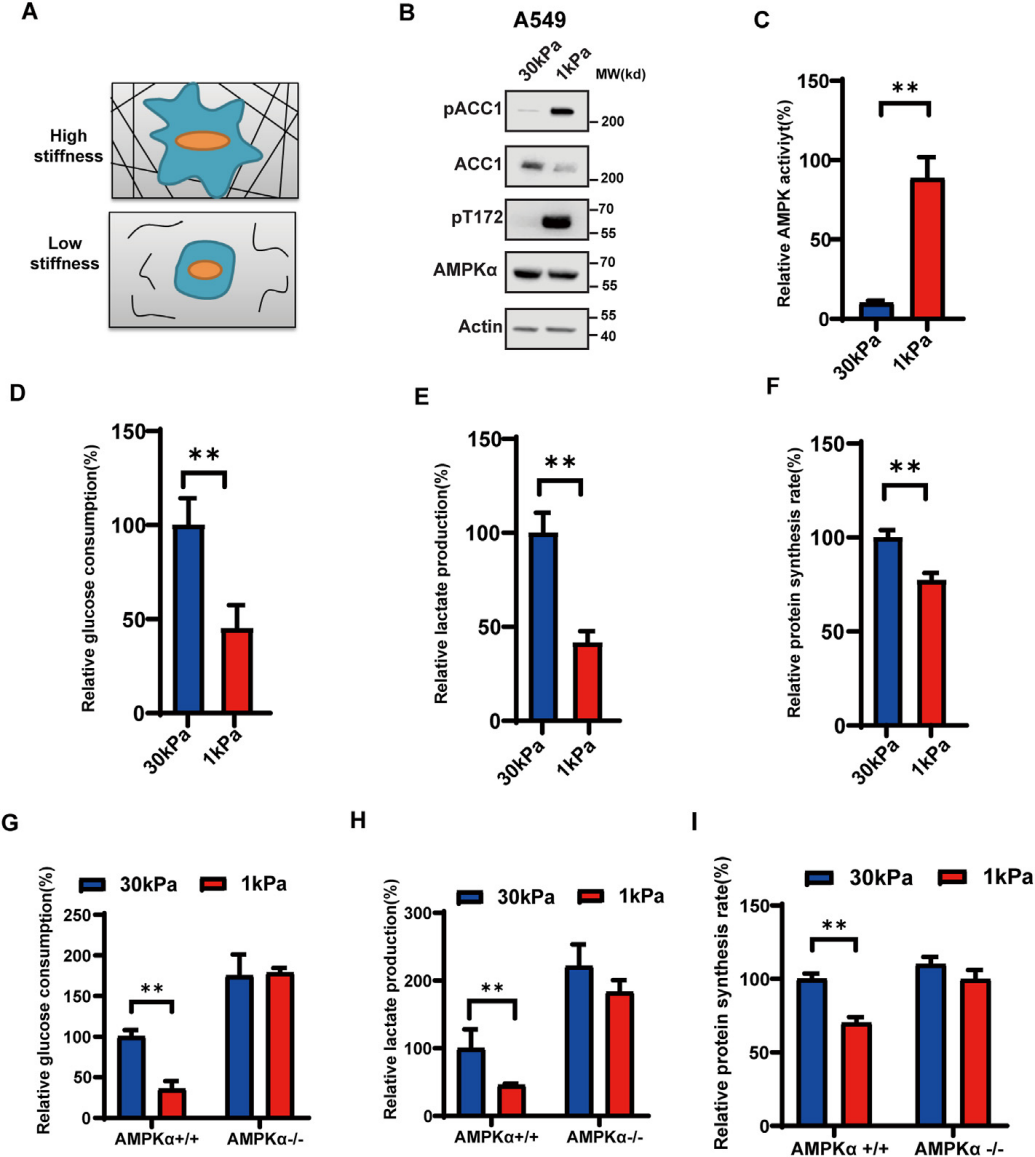
AMPK is activated by low ECM stiffness and mediates cellular metabolic switch. **(A)** A schematic representation of cells grown on ECM with varying stiffness. **(B)** ECM stiffness regulates the phosphorylation of AMPK α and ACC1. Immunoblot analysis of phosphorylation of AMPK (pT172) and ACC1 (pACC1) in cells grown on different stiffness ECMs **(C)** ECM stiffness regulates AMPK activity. AMPK activity in cells grown on hard (30 kPa) and soft (1 kPa) matrices, as determined by immunoprecipitation and activity assay (mean ± SD, *n* = 3). **(D, E)** ECM stiffness regulates aerobic glycolysis. Cells were grown on hard (30 kPa) and soft (1 kPa) matrices. Effects of ECM stiffness on glucose consumption (D) and lactate production (E) in cells were measured. Error bars represent the SD of the mean for three replicates (^∗∗^*P* < 0.01). **(F)** ECM stiffness regulates protein synthesis. Cells were grown on hard (30 kPa) and soft (1 kPa) matrices. Effects of ECM stiffness on glucose consumption (D) and lactate production (E) in cells, as measured by standard assays. Error bars represent the SD of the mean for three replicates (^∗∗^*P* < 0.01). **(G, H)** Effects of ECM stiffness on glucose consumption (G) and lactate production (H) in AMPKα^+/+^ and AMPKα^−/−^ MEF cells grown on hard (30 kPa) and soft (1 kPa) matrices. **(I)** Effects of ECM stiffness on protein synthesis rate in AMPKα^+/+^ and AMPKα^−/−^ MEF cells grown on hard (30 kPa) and soft (1 kPa) matrices.

**Figure S1:**
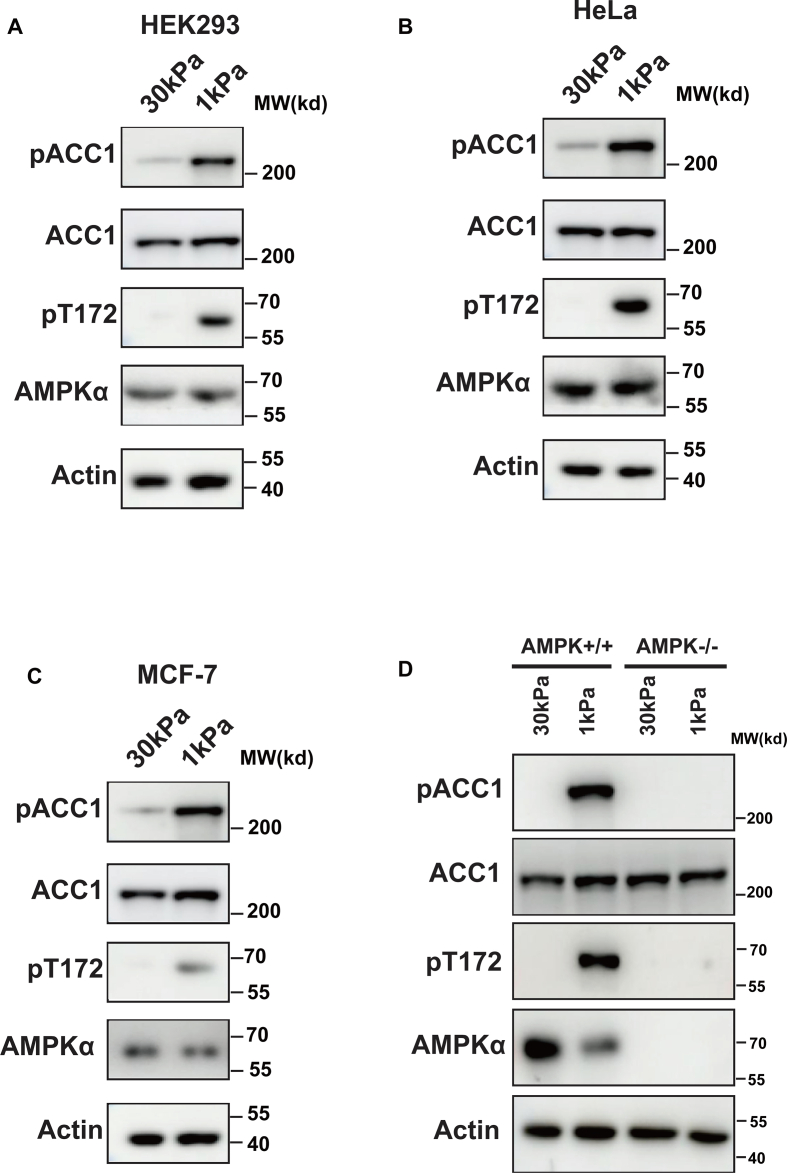
AMKP activity is sensitive to ECM stiffness in various cell lines.

The mechanical property of ECM plays an important role in regulating cellular behaviors such as proliferation, survival, and differentiation. This process is responsible for regulating the ability of cells to develop forces through their contractile actomyosin cytoskeleton and to mature focal adhesions.^32^, ^33^, ^34^ To investigate the effect of ECM stiffness on AMPK activation, we grew cells on different stiffness matrices (Fig. 1A). We found that cells grown on low-stiffness ECMs had higher AMPK activity (Fig. 1B), as indicated by increased AMPK-α phosphorylation at the Thr172 site as well as the AMPK downstream target, acetyl-coenzyme A carboxylase (ACC) (Fig. 1C). Similar activation was observed in the HEK293 cell line and several other epithelial cancer cells, such as HeLa (human cervix epithelial) and MCF7 (human breast epithelial) (Fig. S1A–C). This suggests that AMPK activity may be regulated by ECM stiffness in various epithelial cancers, however further investigation is warranted. Additionally, low ECM stiffness induced inhibition of aerobic glycolysis, including reduced glucose consumption (Fig. 1D), lactate production (Fig. 1E), and protein synthesis rate (Fig. 1F). To confirm that these metabolic effects were mediated by AMPK activation, we knocked out AMPKα1/2 in MEF cells. We found that AMPKα1/2 silencing blocked the ECM stiffness-induced ACC1 phosphorylation (Fig. S1D) and metabolic switch (Fig. 1G–I). These findings suggest that AMPK is activated by low ECM stiffness and mediates a metabolic shift under different ECM stiffness conditions.

### ECM stiffness regulates AMPK activity independent of LKB1 and CaMKKβ

**Figure 2:**
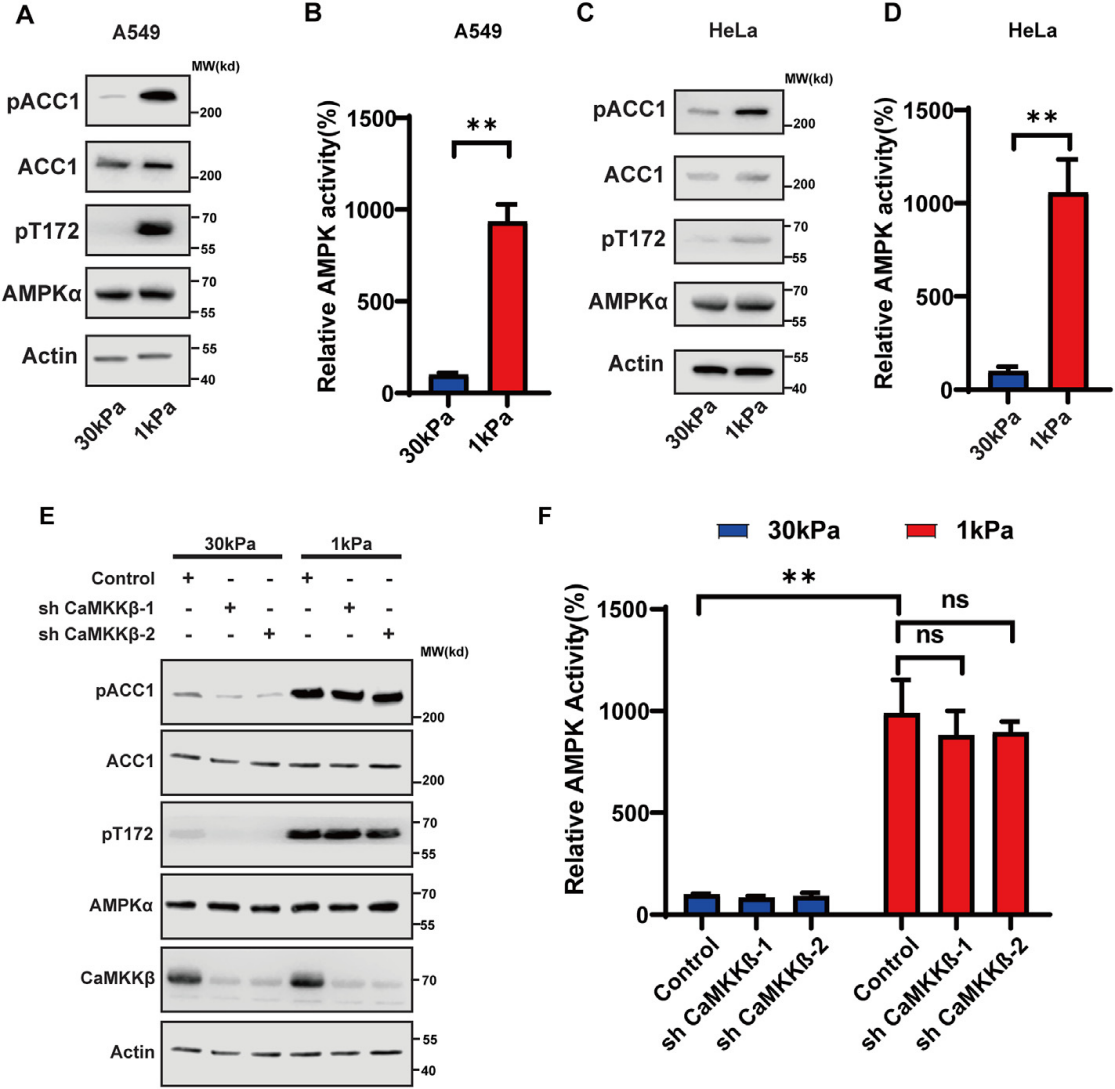
Low ECM stiffness induces AMPK activation independent of CaMKKβ and LKB1. **(A, B)** ECM stiffness regulates AMPK activity in A549 cells (lacking LKB1). A549 cells were grown on hard (30 kPa) and soft (1 kPa) matrices and cell lysates were blotted with the indicated antibodies (A) or used for AMPK activity assay (B). **(C, D)** ECM stiffness regulates AMPK activity in HeLa cells (lacking LKB1). HeLa cells were grown on hard (30 kPa) and soft (1 kPa) matrices and cell lysates were blotted with the indicated antibodies (C) or used for AMPK activity assay (D). **(E, F)** ECM stiffness regulates AMPK activity independent of CaMKKβ and LKB1. A549 cells expressing the indicated CaMKKβ shRNA were grown on hard (30 kPa) and soft (1 kPa) matrices and cell lysates were blotted with indicated antibodies (E) or used for AMPK activity assay (^∗∗^*P* < 0.01; ns, not significant) (F).

AMPK is the key sensor of energy stress and is activated by increased ADP/ATP or AMP/ATP ratios which induce AMPKα phosphorylation by LKB1.^9^^,^^35^^,^^36^ Therefore, we posed the following question: is LKB1 responsible for AMPK activation by mechanical cues? Several pieces of evidence indicated this is not the case. First, we noted that the ADP/ATP ratio was not increased upon treatment with C3 or at low ECM stiffness conditions (data not shown). Second, C3 treatment and low ECM stiffness conditions induced AMPKα phosphorylation and activation in A549 and HeLa cells (Fig. 2A–D), which do not have endogenous LKB1 expression.^9^^,^^16^ The other kinase that mediates AMPKα phosphorylation is CaMKKβ, which can be activated by increased cellular calcium concentration.^15^, ^16^, ^17^ To assess whether AMPK activation is mediated by CaMKKβ, we silenced CaMKKβ in A549 cells. The expression of shRNAs targeting CaMKKβ reduced total CaMKKβ protein expression, but CaMKKβ silencing did not affect AMPK phosphorylation induced by the low ECM stiffness condition (Fig. 2E, F). Taken together, these data suggest that ECM stiffness controls AMPK activation through unknown kinase(s) other than LKB1 and CaMKKβ.

### AMPK senses cytoskeletal tension

**Figure S2:**
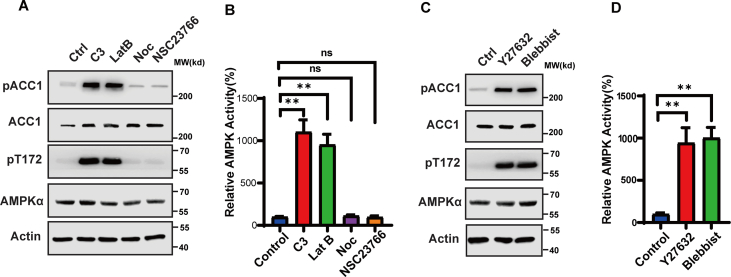
AMPK activation is controlled by Rho and the tension of the actin cytoskeleton, independent of CaMKKβ and LKB1.

The small GTPase Rho regulates the formation of actin bundles, stress fibers, and tensile actomyosin structures. ECM stiffness controls the activation of Rho.^37^, ^38^, ^39^, ^40^ We considered whether Rho and the actin cytoskeleton are involved in regulating AMPK activation. As shown in Figure S2A and 2B, the inhibition of Rho and the actin cytoskeleton activated AMPK. As a control, we found that inhibition of guanine nucleotide exchange factor Rac1-GEF by NSC23766 or disruption of microtubules by nocodazole did not affect AMPK activity (Fig. S2A, 2B).

When ECM stiffness increases, cells adjust the tension and organization of their stress fibers by increasing pulling forces against the ECM to accompany cell spreading.^40^, ^41^, ^42^ We investigated whether ROCK and myosin are involved in regulating AMPK activation by ECM stiffness. We found that after the inhibition of ROCK with Y27632 and non-muscle myosin with blebbistatin, cytoskeletal tension inhibited the phosphorylation of AMPK-α and ACC1 as well as AMPK activation (Fig. S2C, 2D). Notably, the activation of AMPK caused by these inhibitors is a very early event that occurs within 2 h, excluding the possibility that the activation of AMPK might be caused by the destabilization of stress fibers. Together, these results indicate that AMPK senses cytoskeletal tension at different ECM stiffness conditions.

### Hippo kinases phosphorylate AMPKα Thr172 *in vitro* and in cells

**Figure S3:**
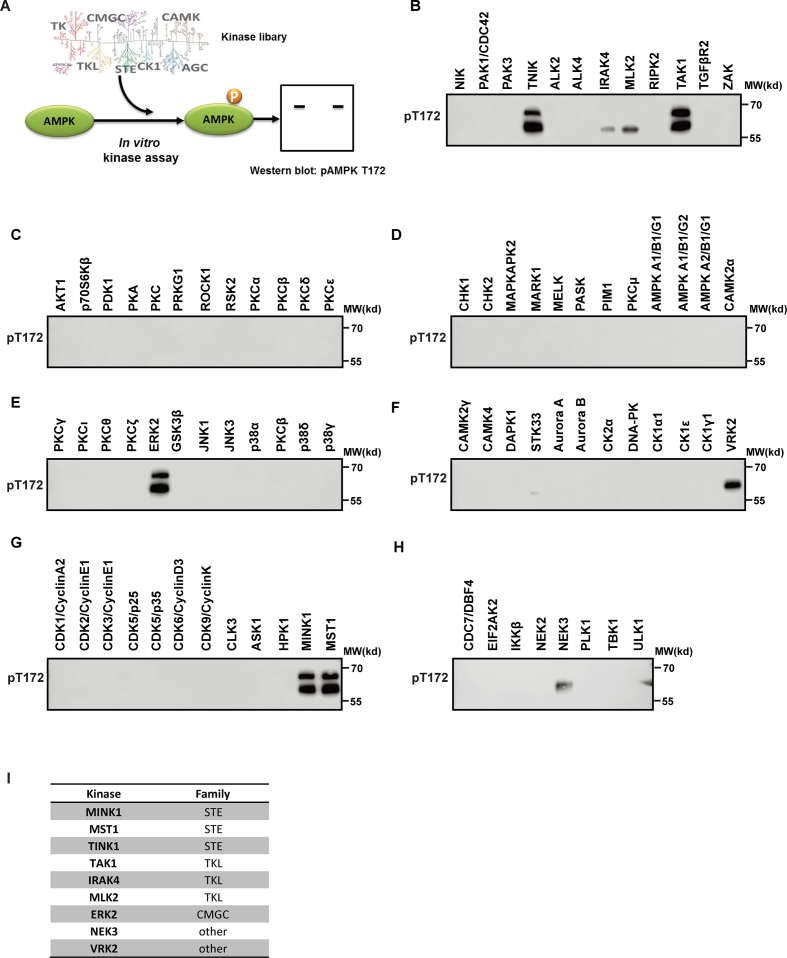
Identification of AMPKα-activating kinases.

Considering the indispensable role of AMPK in cell metabolism regulation, identifying the kinases that activate AMPK independently of LKB1/CaMKKβ is crucial for understanding the signal transduction mechanism underlying ECM stiffness. Therefore, we screened a kinase library to identify candidate kinases that can directly phosphorylate AMPKα Thr172. A full-length human AMPKα1 was purified from *Escherichia coli* and used as a substrate for *in vitro* kinase assays. We screened a purified kinase set comprising 80 kinases from different subfamilies (Fig. S3A). Through this approach, we identified nine kinases that can efficiently phosphorylate AMPKα Thr172. These included MINK1 (MAP4K6), MST1, TINK1 (MAP4K7), TAK1, IRAK4, MLK2, ERK2, NEK3, and VRK2 (Fig. S3B–I).

MST1, MINK1 (MAP4K6), and TINK1 (MAP4K7) belong to the STE20-like kinase family, which consists of MST1/2 kinases and MAP4K. These kinases are highly homologous to one another and identified as core Hippo kinases (MAP4K family kinases and MST1/2), which can be activated by mechanical signals such as low ECM stiffness, high cell density, and cell detachment.^43^, ^44^, ^45^ These findings suggest that the Hippo kinases (MAP4K family kinases and MST1/2) were the most promising candidates for the activation of AMPKα in response to ECM stiffness. Therefore, we focused on these Hippo kinases in this study.

**Figure 3:**
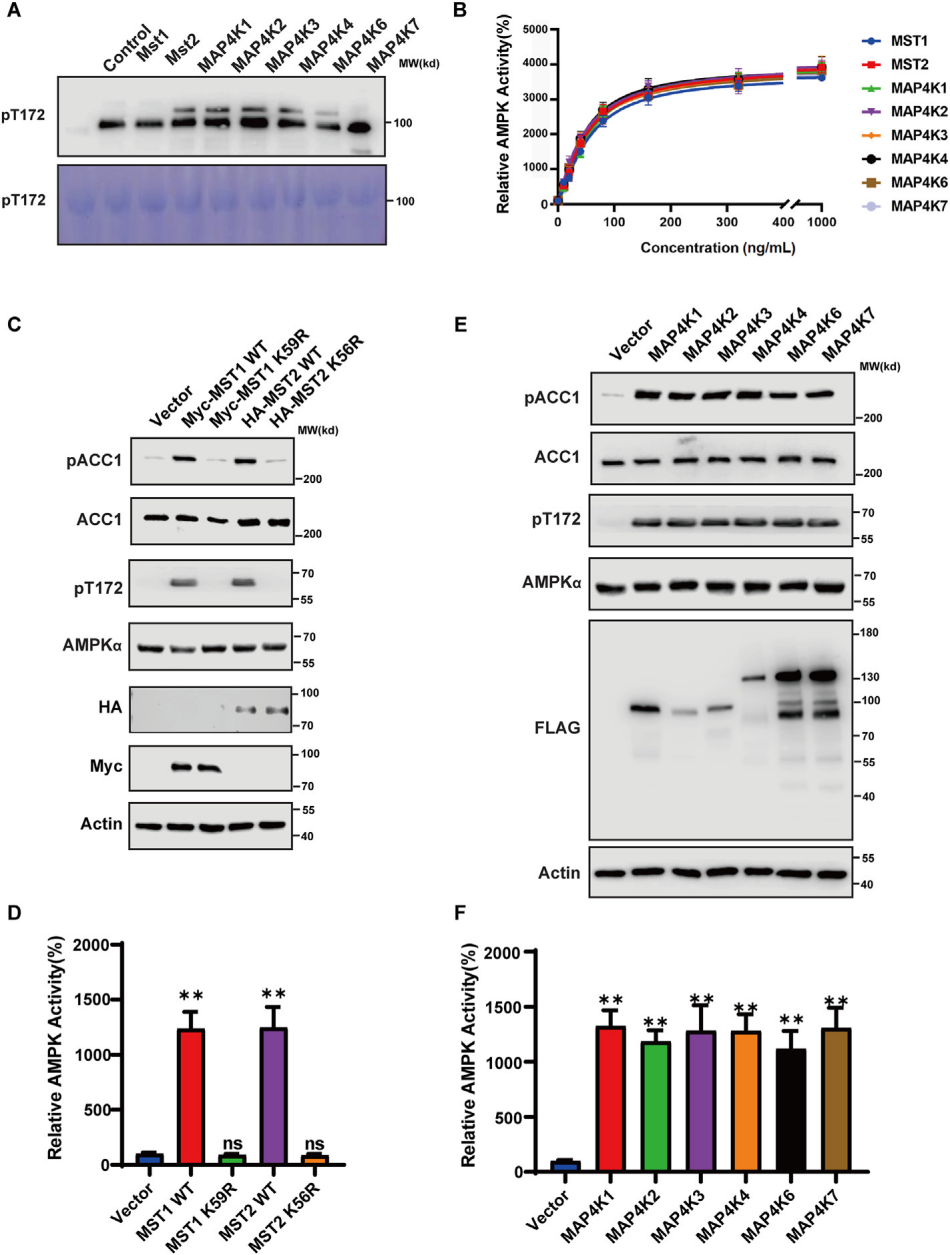
Hippo kinases (MST1/2 and MAP4Ks) phosphorylate AMPK Thr172 *in vitro* and in cells. **(A)** Phosphorylation of AMPKα1 by Hippo kinases. Approximately 0.2 μg of purified Hippo kinases (MST1, MST2, MAP4K1, MAP4K2, MAP4K3, MAP4K4, MAP4K6, and MAP4K7) were incubated with 1 μg of bacterially expressed GST-AMPKα1. Phosphorylation of the AMPKα1 was detected with the phospho-specific antibody. Coomassie brilliant blue staining was used as a loading control. **(B)** The activity of the AMPK complex (0.05 μg) following phosphorylation with varying amounts of Hippo kinases was determined using the SAMS peptide assay. **(C)** Overexpression of wild-type MST1/2, but not kinase-dead mutants (MST1 K59R or MST2 K56R), induced AMPKα Thr172 phosphorylation. HEK293 cells were transfected with the kinase expression plasmids; phosphorylation of AMPK (pT172) and ACC1 (pACC1) was analyzed using immunoblotting. **(D)** Overexpression of wild-type MST1/2 but not kinase-dead mutants (MST1 K59R or MST2 K56R) induced AMPK activation. HEK293 cells were transfected with the kinase expression plasmids, immunoprecipitated with anti-AMPKα antibody, and AMPK activity in immunoprecipitates was measured (^∗∗^*P* < 0.01; ns, not significant). **(E)** Overexpression of MAP4K kinases induced AMPKα Thr172 phosphorylation. HEK293 cells were transfected with expression plasmids for different MAP4K family kinases, and phosphorylation of AMPK (pT172) and ACC1 (pACC1) was analyzed by immunoblotting. **(F)** Overexpression of MAP4K family kinases induced AMPK activation. HEK293 cells were transfected with the expression plasmids for different MAP4K family kinases, immunoprecipitated with anti-AMPKα antibody, and AMPK activity in immunoprecipitates was measured (^∗∗^*P* < 0.01).

To test whether AMPKα can be directly phosphorylated by these Hippo kinases, we performed *in vitro* kinase assays with purified Hippo kinases. We found that all eight Hippo kinases phosphorylate AMPKα to a similar extent (Fig. 3A). Notably, AMPKα alone can be readily phosphorylated by Hippo kinases, independent of the β and γ subunits.

To further study the role of Hippo kinases in activating AMPK, we performed a sequential kinase assay of Hippo kinases–AMPKα–SAMS *in vitro*. We found that all Hippo kinases enhanced AMPK activity as determined by SAMS peptide phosphorylation (Fig. 3B). These results, combined with the previous findings of the *in vitro* kinase assays, demonstrate that Hippo kinases can directly activate AMPK. To test whether Hippo kinases regulate AMPK signaling in cells, we transfected plasmids expressing these Hippo kinases into HEK293 cells. We found that the expression of wild-type MST1 and MST2 individually induced activation of AMPK and promoted phosphorylation on AMPK-α and ACC, while the expression of kinase-dead mutants (MST1K56R, MST2K59R) did not (Fig. 3C, D). Furthermore, expressing MAP4K family kinases induced AMPKα phosphorylation as well as AMPK activation (Fig. 3E, F), indicating that Hippo kinases can phosphorylate AMPK in cells.

**Figure S4:**
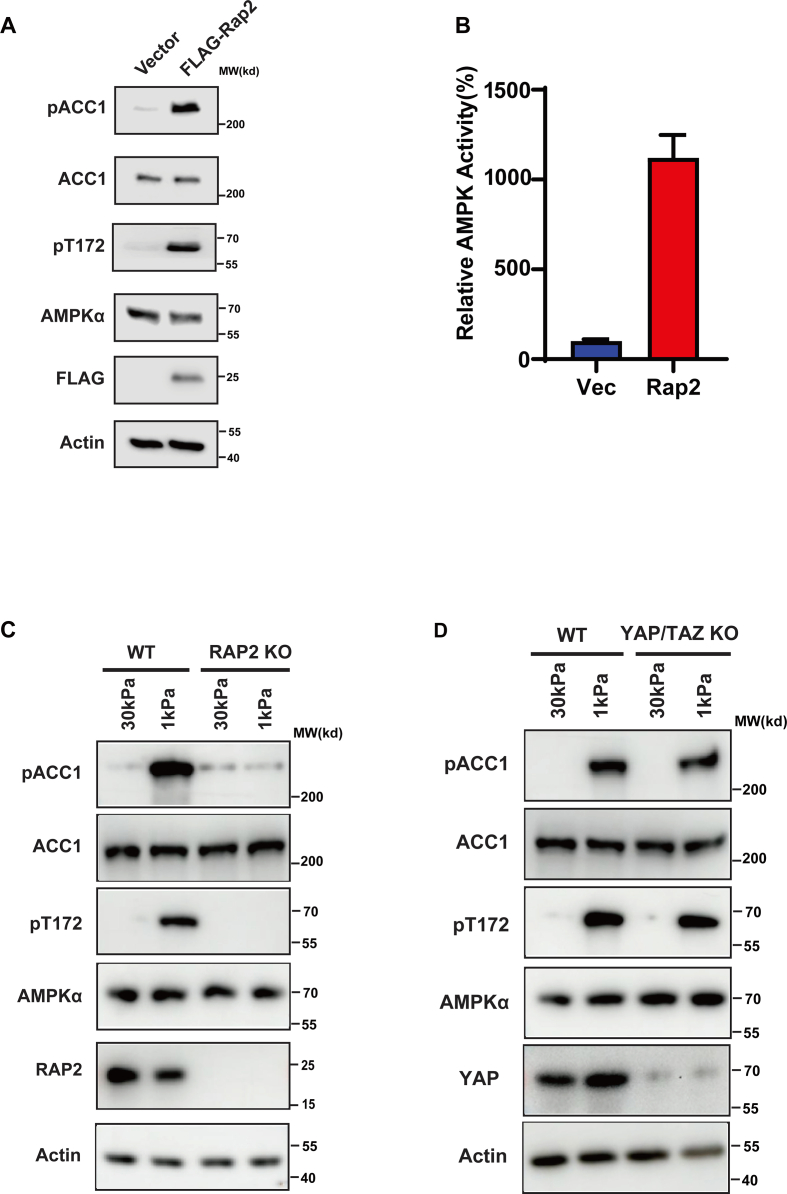
AMPK is activated by RAP2 rather than YAP/TAZ.

Rap2A has recently been reported as an essential intracellular signal transducer that relays ECM stiffness signals to modulate MAP4K family kinase activity.^43^ Remarkably, Rap2A overexpression increased AMPKα Thr172 phosphorylation and activation (Fig. S4A, 4B). In addition, Rap2 KO cells cannot sense ECM stiffness to increase the phosphorylation of AMPKα Thr172 (Fig. S4C). As well-known effectors regulated by ECM stiffness through the Hippo pathway,^46^ YAP/TAZ was blocked to sense mechanical cues by deletion of Rap2.^43^ To further explore the role of YAP/TAZ in the response of AMPK activation to ECM stiffness, we performed YAP/TAZ KO in cells on different mechanical materials. Notably, we found that the phosphorylation of AMPKα Thr172 stimulated by ECM stiffness did not dependent on YAP/TAZ (Fig. S4D). Together, we confirmed that the Rap2–Hippo kinases axis plays a role in regulating AMPK activation.

### Hippo kinases mediate AMPK activation and metabolic switch in response to low ECM stiffness

**Figure 4:**
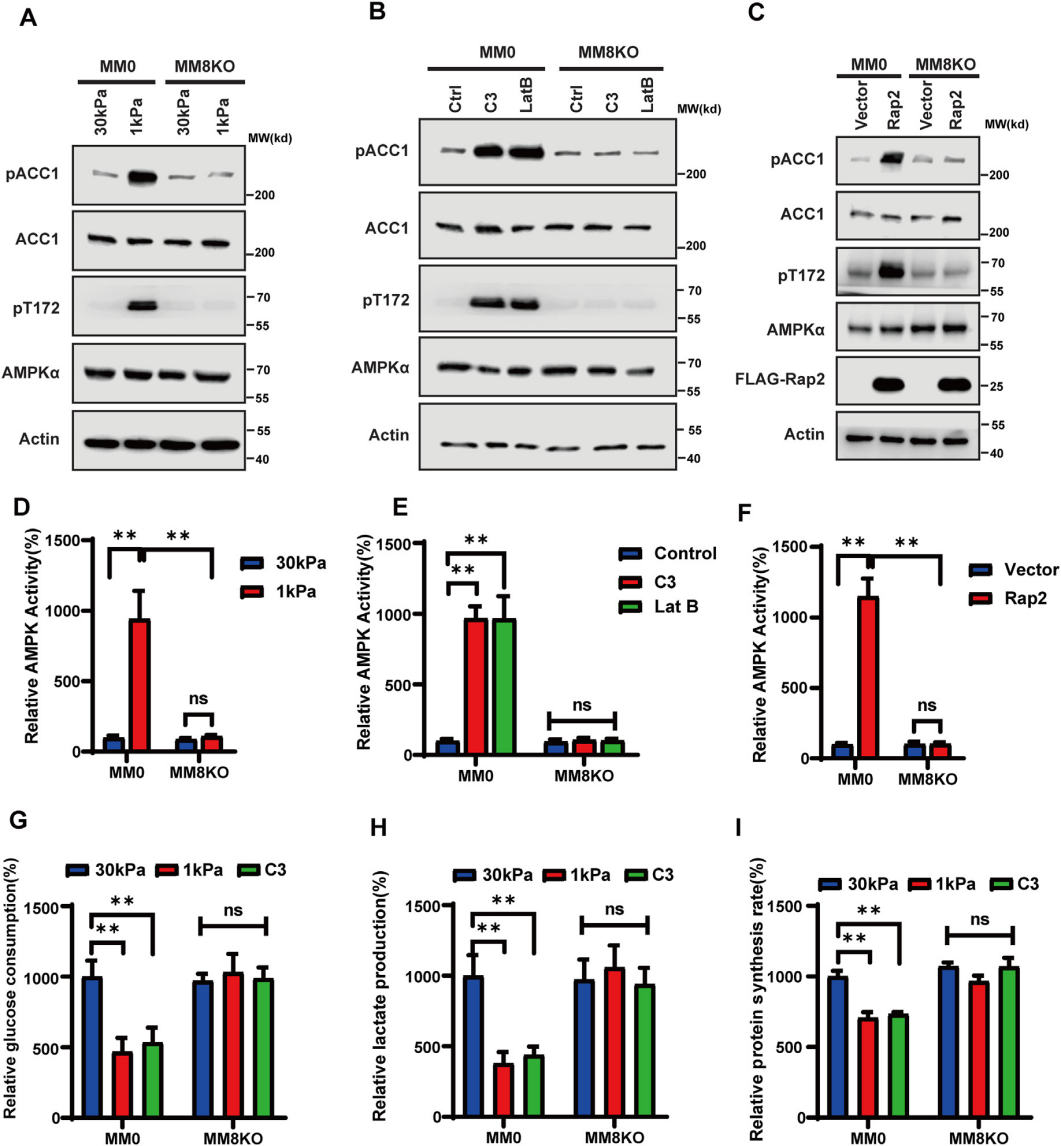
Hippo kinases mediate AMPK activation and cellular metabolic switch in response to low ECM stiffness. **(A)** Hippo kinases mediate phosphorylation of AMPK (pT172) and ACC1 (pACC1) under the low ECM stiffness condition. Wild type (MM0) and Hippo kinases knockout (MM-8KO) HEK293 cells were grown on hard (30 kPa) or soft (1 kPa) matrices, and cell lysates were blotted with the indicated antibodies. **(B)** Hippo kinases mediate AMPK activation under the low ECM stiffness condition. Wild type (MM0) and Hippo kinases knockout (MM-8KO) HEK293 cells were grown on hard (30 kPa) or soft (1 kPa) matrices and cell lysates were used for AMPK activity assay **(C)** Hippo kinases mediate phosphorylation of AMPK (pT172) and ACC1 (pACC1) by Rho and F-actin inhibition. Wild type (MM0) and Hippo kinases knockout (MM-8KO) HEK293 cells grown on Petri dishes were treated with Rho inhibitor C3 (5 μg/mL) and the F-actin inhibitor latrunculin B (Lat. B, 1 μM); cell lysates were blotted with the indicated antibodies. **(D)** Hippo kinases mediate AMPK activation by Rho and F-actin inhibition. Wild type (MM0) and Hippo kinases knockout (MM-8KO) HEK293 cells grown on Petri dishes were treated with Rho inhibitor C3 (5 μg/mL) and the F-actin inhibitor latrunculin B (Lat. B, 1 μM); cell lysates were used for AMPK activity assay (^∗∗^*P* < 0.01; ns, not significant). **(E)** Hippo kinases mediate phosphorylation of AMPK (pT172) and ACC1 (pACC1) by Rap2 overexpression. Wild type (MM0) and Hippo kinases knockout (MM-8KO) HEK293 cells grown on Petri dishes were transfected with Vector or FLAG-Rap2a; cell lysates were blotted with indicated antibodies (^∗∗^*P* < 0.01; ns, not significant). **(F)** Hippo kinases mediate AMPK activation by Rap2 overexpression. Wild type (MM0) and Hippo kinases knockout (MM-8KO) HEK293 cells grown on Petri dishes were transfected with Vector or FLAG –Rap2a; cell lysates were used for AMPK activity assay (^∗∗^*P* < 0.01; ns, not significant). **(G, H)** Glucose consumption (G) and lactate production (H) of MM0 and MM-8KO cells grown on hard (30 kPa) and soft (1 kPa) matrices or treated with Rho inhibitor C3 (5 μg/mL) (^∗∗^*P* < 0.01; ns, not significant). **(I)** Protein synthesis rate of MM0 and MM-8KO cells grown on hard (30 kPa) and soft (1 kPa) matrices or treated with Rho inhibitor C3 (5 μg/mL) (^∗∗^*P* < 0.01; ns, not significant).

To determine the role of Hippo kinases in the AMPK pathway *ex vivo*, we utilized the Hippo kinase KO cell line MM-8KO.^43^ We found that deletion of all Hippo kinases severely diminished phosphorylation of AMPK and ACC1 induced by low ECM stiffness (Fig. 4A), Lat B/C3 treatment (Fig. 4B), and Rap2 overexpression (Fig. 4C). Furthermore, we observed that AMPK activation by low ECM stiffness (Fig. 4D), Lat B/C3 treatment (Fig. 4E), and Rap2 overexpression (Fig. 4F) was also significantly blocked in MM-8KO cells. Together, these data demonstrate that low ECM stiffness induces AMPK activation through Hippo kinases.

Given that Hippo kinases mediate AMPK activation by low ECM stiffness, we further examined the effect of Hippo kinases on the metabolic switch under these circumstances. In line with the role of Hippo kinase dependency on AMPK activation, we observed that KO of Hippo kinase rescued glucose consumption (Fig. 4G), lactate production (Fig. 4H), and protein synthesis (Fig. 4I) upon C3 treatment or growth on soft matrix. This indicated that the Hippo kinases mediated the metabolic switch in response to different ECM stiffness conditions.

### Association of pAMPK and α-SMA expression in patients with pancreatic cancer with their survival

**Figure 5:**
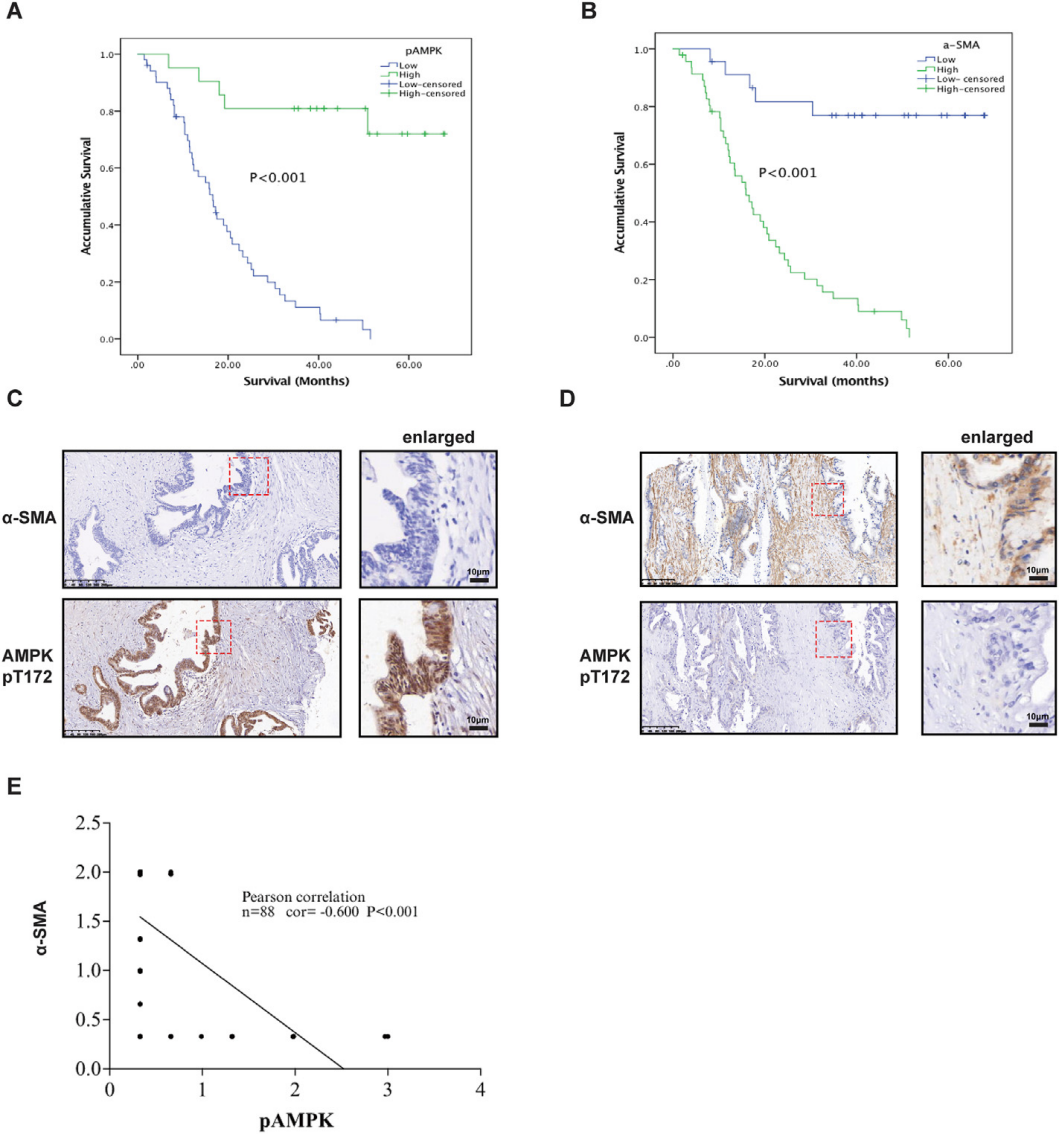
Association between pAMPK and α-SMA expression level and survival in patients with pancreatic cancer. **(A, B)** Survival of PDAC patients with various levels of pAMPK and α-SMA. Kaplan–Meier survival plots show that lower expression of pAMPK (A) or higher expression of α-SMA results in a worse survival outcome in PDAC patients. **(C, D)** Representative staining of pAMPK and α-SMA in PDAC. Highlighted areas are enlarged on the right. **(E)** Correlation between pAMPK and α-SMA expression in tumor tissues.

**Figure S5:**
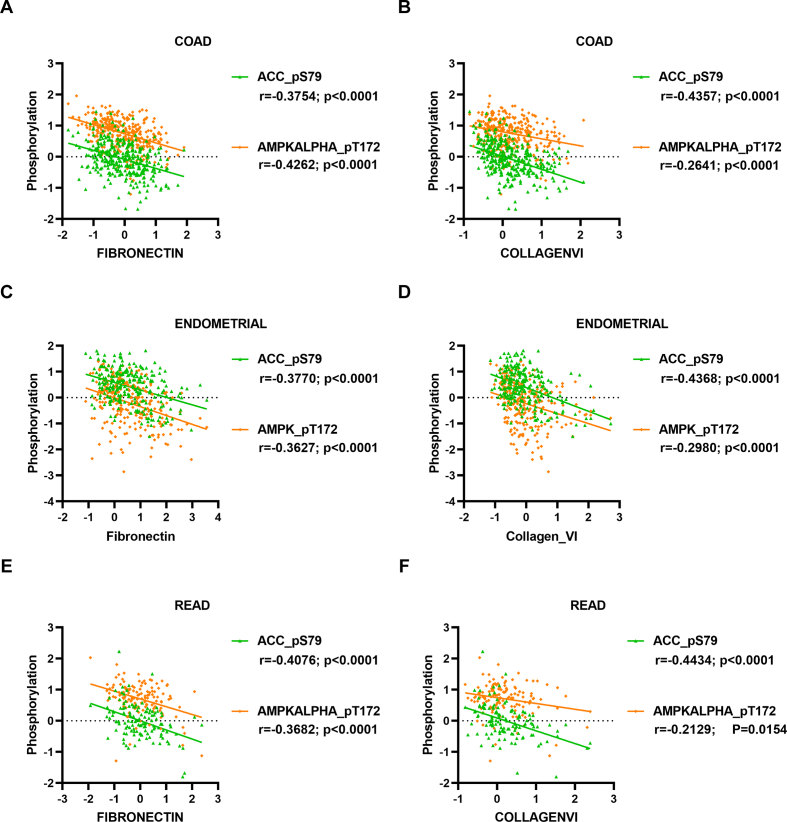
Correlation of AMPK activity with ECM context.

PDAC is characterized by a dense fibrotic stroma that is surrounded by an ECM, and the accumulation of highly abundant ECM proteins in PDAC exerts mechanical properties on tumor cells.^47^^,^^48^ Given that high expression of ECM proteins leads to changes in the physical properties of the ECM and increases its stiffness, we used α-SMA expression as a marker of high ECM stiffness in PDAC. From multivariate survival analyses, we found that pAMPK and α-SMA expression in PDAC were independent prognostic factors for patients with pancreatic cancer (Table S1). PDAC patients with low α-SMA expression or high pAMPK expression in cancer tissues had a significant improvement in OS (Fig. 5A, B). Representative images of high and low/no staining of pAMPK and α-SMA in PDAC are shown in Figure 5C and D. The level of pAMPK was mostly absent or low (73.4%) in most PDAC patients, and only 26.6% of patient specimens showed a high level of pAMPK staining. Conversely, the level of α-SMA (68.8%) was high in most PDAC patients. Interestingly, we found that pAMPK expression levels were negatively correlated with α-SMA (*r* = −0.600, *P* < 0.001) in the PDAC tissues (Fig. 5E). Moreover, this negative correlation between pAMPK and ECM protein was further confirmed in multiple cancers by RPPA data from TCGA (Fig. S5A–F). These results further confirmed that AMPK activity is negatively affected by ECM stiffness.

### Targeting the ECM stiffness–Hippo kinases–AMPK and low glucose–LKB1–AMPK signaling pathways simultaneously can inhibit the growth of PDAC

**Figure 6:**
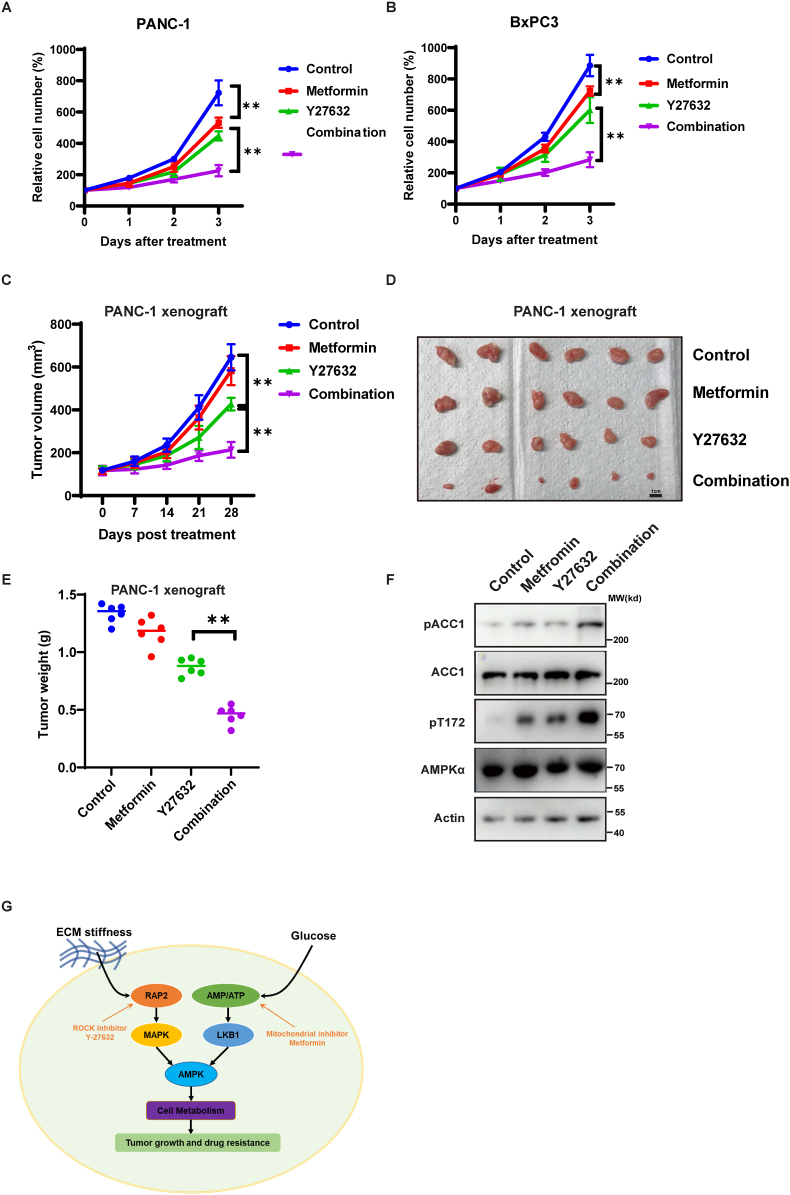
A combination of metformin and ROCK inhibitor Y27632 inhibits pancreatic ductal adenocarcinoma growth. **(A, B)** Growth curve of PANC1 (A) and BxPC3 cells grown on hard (30 kPa) matrix treated with mitochondria inhibitor metformin (10 μM), ROCK inhibitor Y27632 (10 μM), or combination of metformin and Y27632. **(C)** 5 × 10^6^ PANC1 cells were embedded in 200 μL 9.0 kPa hyaluronan-based gel and were subcutaneously injected into nude mice. Mice were treated with metformin (200 mg/kg in drinking water), Y27632 (30 mg/kg, every 3 d), or a combination as indicated. Tumor growth was measured at the indicated times after treatment. *n* = 6 for each group; ^∗∗^*P* < 0.01, by two-way ANOVA. **(D)** Images of tumors as described in (C) on day 28 of treatment. **(E)** Tumor weight of mice as described in (C) at day 28 of treatment. ^∗∗^*P* < 0.01, by two-way ANOVA. **(F)** Phosphorylation of AMPK (pT172) and ACC1 (pACC1) in tumors as described in (C). Immunoblot analysis of phosphorylation of AMPK (pT172) and ACC1 (pACC1) in tumor tissues as described in (C). **(G)** A proposed working model. Both ECM stiffness–RAP2–MAPK signaling and low glucose–LKB1 signaling regulate AMPK activation. Simultaneously targeting the ECM stiffness–MAPK–AMPK signaling and low glucose–LKB1–AMPK signaling pathways fully activates AMPK, thus inhibiting PDAC tumor growth.

Given that both the ECM stiffness–Hippo kinases and low glucose–LKB1 signaling pathways control AMPK activation, we proposed that simultaneously targeting these two pathways could fully activate AMPK and inhibit PDAC growth. To test this, we utilized ROCK inhibitor Y-27632 to activate Hippo kinase–AMPK signaling, and the mitochondrial inhibitor metformin to activate the LKB1–AMPK signaling. As shown in Figure 6A and B, the combination of Y-27632 and metformin significantly inhibited PANC-1 and BxPC3 growth. We also tested this combination treatment in a PANC-1 xenograft mice model and found that it dramatically inhibited PANC-1 xenograft tumor growth (Fig. 6C–E) and AMPK activation (Fig. 6F). These results suggest that simultaneously activating the Hippo kinases–AMPK and LKB1–AMPK signaling pathways could be an attractive therapeutic strategy for PDAC treatment (Fig. 6G).

## Discussion

**Figure S6:**
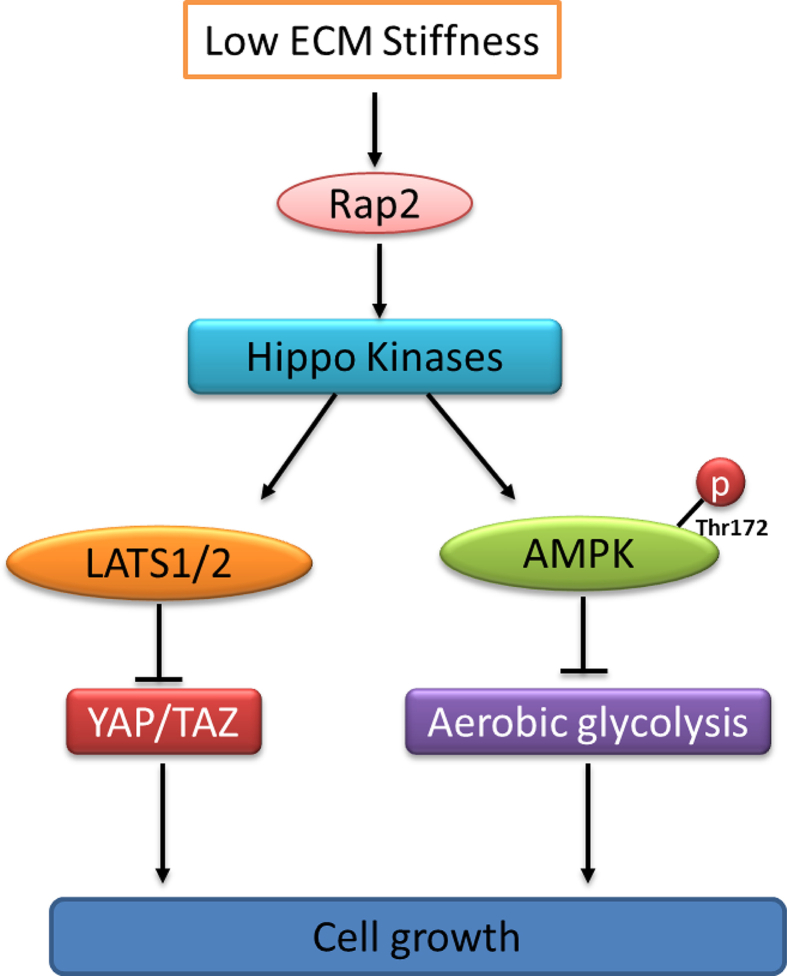
Model for coordinated regulation of cell metabolism and cell growth by ECM stiffness.

Our data has identified a novel modality of AMPK activity controlled by Hippo kinases, in addition to the well-known LKB1–AMPK pathway. This is similar to the Rap2–Hippo kinase–LATS1/2 pathway (Fig. S6). Both pathways require inhibition of the small GTPase Rho activity and disruption of the actomyosin cytoskeleton, and they share the same upstream activating kinases (Hippo kinases). KO of the eight Hippo kinases blocked the activation of AMPK and LATS1/2 by low ECM stiffness. LATS1/2 play key roles in cell proliferation through YAP/TAZ-mediated transcription,^49^ while AMPK controls the metabolic switch between anabolism and catabolism.^4^ Therefore, these two pathways might coordinate with each other for proper cell proliferation through the common Rap2–Hippo kinase pathway.

In this study, we have shown that AMPK is regulated by ECM stiffness. In addition, energy stress such as low extracellular glucose can modulate the ECM components and decrease ECM stiffness through AMPK.^50^, ^51^, ^52^ This may create an extracellular feedforward loop between ECM stiffness and energy stress which ensures proper AMPK activity and cellular metabolic status according to the cellular microenvironment. Disruption of this feedforward loop may lead to tissue stiffening and interrupted metabolic balance during the pathological progression of diseases such as aging, cancer, fibrosis, and cardiovascular disease, which are accompanied by decreased AMPK activity.^50^^,^^53^^,^^54^ Therefore, activating this feedforward loop, by preventing or reversing tissue stiffening, thus interrupting the cellular response, could be a therapeutic approach with potential clinical application.

## Conclusions

AMPK is an ECM stiffness-sensitive protein kinase. In this study, a novel ECM stiffness–Rap2–Hippo kinases–AMPK pathway was established. Disrupting this pathway, by preventing or reversing tissue stiffening or interrupting the cellular response, or by activation of AMPK, could be a therapeutic approach with clinical potential.

## Author contributions

Y.L., M.D., and Z.L. designed and interpreted the experiments and wrote the manuscript. X.X., Y.F., S.N., and M.D. performed the experiments.

## Conflict of interests

The authors declare no competing financial interests.

## Funding

This work was supported in part by grants from the 10.13039/501100001809Natural Science Foundation of China (No. 82272757), CAMS Innovation Fund for Medical Sciences (No. 2021-I2M-1-067), Non-profit Central Research Institute Fund of 10.13039/501100005150Chinese Academy of Medical Sciences (No. 2021-RC310-013), Mayo Foundation, and Beijing Hope Run Special Fund of 10.13039/501100019870Cancer Foundation of China (No. LC2021R02).

## Data availability

The data that support the findings of this study are available from the corresponding author upon reasonable request.
