# Supplementary material for: Regulation of AMPK activation by extracellular matrix stiffness in pancreatic cancer
Source: Genes Dis. 2023 Jul 14;11(3):101035. doi: 10.1016/j.gendis.2023.05.022 (PMC10825306; doi:10.1016/j.gendis.2023.05.022)
Supplement: Multimedia component 1 [file mmc1.docx]

**Supplementary table 1 Survival analysis for prognostic factors of OS**

| **Characteristics** | | **Univariate analysis** | | **Multivariate analysis** | |
| --- | --- | --- | --- | --- | --- |
|  |  | HR (95% CI) | *P* | HR (95% CI) | *P* |
| Gender | Female (Ref) |  |  |  |  |
|  | Male | 0.462 (0.174-1.225) | 0.120 |  |  |
| Age, years (continuous) | | 0.950 (0.896-1.007) | 0.084 |  |  |
| CA19-9 | ≤ 37 (Ref) |  |  |  |  |
|  | > 37 | 0.981(0.299-3.221) | 0.975 |  |  |
| CEA | ≤ 5 (Ref) |  |  |  |  |
|  | > 5 | 0.403(0.124-1.305) | 0.129 |  |  |
| Tumor differentiation | I/II (Ref) |  |  |  |  |
|  | III | 4.432 (1.590-12.439) | 0.004 |  | NA |
| Tumor size, cm (continuous) | | 1.022 (0.761-1.373) | 0.883 |  |  |
| Lymph node metastasis | No (Ref) |  |  |  |  |
|  | Yes | 3.669 (1.205-11.173) | 0.022 |  | NA |
| Nerve invasion | No (Ref) |  |  |  |  |
|  | Yes | 5.200 (1.490-18.149) | 0.010 |  | NA |
| Vascular invasion | No (Ref) |  |  |  |  |
|  | Yes | 0.836 (0.072-9.685) | 0.886 |  |  |
| TNM stage | I (Ref) |  |  |  |  |
|  | II | 6.133 (0.519-72.524) | 0.150 |  |  |
|  | III | 2.857 (0.215-37.990) | 0.426 |  |  |
| Chemotherapy | No (Ref) |  |  |  |  |
|  | Yes | 0.145 (0.044-0.481) | 0.002 |  | NA |
| Radiotherapy | No (Ref) |  |  |  |  |
|  | Yes | 0.611 (0.095-3.912) | 0.603 |  |  |
| Operation type | PD (Ref) |  |  |  |  |
|  | DP | 0.880 (0.333-2.324) | 0.796 |  |  |
| Operation time | ≤ 3 h (Ref) |  |  |  |  |
|  | > 3 h | 0.617 (0.211-1.802) | 0.377 |  |  |
| Blood loss | ≤ 300 ml (Ref) |  |  |  |  |
|  | > 300 ml | 1.628 (0.475-5.577) | 0.438 |  |  |
| Transfusion | No (Ref) |  |  |  |  |
|  | Yes | 0.673 (0.147-3.073) | 0.609 |  |  |
| pAMPK | Low |  |  |  |  |
|  | High | 0.034(0.009-0.133) | <0.001 | **0.155(0.013-0.920)** | **0.037** |
| a-SMA | Low |  |  |  |  |
|  | High | 52.8(11.401-244.53) | <0.001 | **29.941(1.079-83.92)** | **0.045** |
