# Supplementary material for: Regulation of AMPK activation by extracellular matrix stiffness in pancreatic cancer
Source: Genes Dis. 2023 Jul 14;11(3):101035. doi: 10.1016/j.gendis.2023.05.022 (PMC10825306; doi:10.1016/j.gendis.2023.05.022)
Supplement: Multimedia component 2 [file mmc2.docx]

Figure S1. AMKP activity is sensitive to ECM stiffness in various cell lines.

(A-C) ECM stiffness regulates phosphorylation of AMPK α and ACC1. Immunoblot analysis of phosphorylation of AMPK (pT172) and ACC1 (pACC1) in HEK293 cells(A), HeLa cells（B）and MCF7 cells(C) grown on different ECM stiffness.

(D) Phosphorylation of AMPK (pT172) and ACC1 (pACC1) in WT or AMPK-/- MEFs. Immunoblot analysis of phosphorylation of AMPK (pT172) and ACC1 (pACC1) in WT or AMPK-/- MEFs cultured on different ECM stiffness.

Figure S2. AMPK activation is controlled by Rho and tension of the actin cytoskeleton, independent of CaMKKβ and LKB1.

(A) Phosphorylation of AMPK (pT172) and ACC1 (pACC1) in cells treated with Rho inhibitor C3 (5μg/ml), the F-actin inhibitor latrunculin B (Lat.B, 1μM), the Rac-GEFs inhibitor NSC23766 (100μM) or the microtubule inhibitor nocodazole (Noc, 100ng/ml).

(B) AMPK activity in cells treated with Rho inhibitor C3 (5μg/ml), the F-actin inhibitor latrunculin B (Lat.B, 1μM), the Rac-GEFs inhibitor NSC23766 (100μM) or the microtubule inhibitor nocodazole (Noc, 100ng/ml) (**: p < 0.01; ns: no significant).

(C) Phosphorylation of AMPK (pT172) and ACC1 (pACC1) in cells treated with ROCK inhibitor Y27632 (50μM) or the non-muscle myosin inhibitor blebbistatin (Blebbist, 100μM).

(D) AMPK activity in cells treated with ROCK inhibitor Y27632 (50μM) or the non-muscle myosin inhibitor blebbistatin (Blebbist, 100μM) (**: p < 0.01).

Figure S3. Identification of AMPKα-activating kinases.

(A) A scheme of the in vitro kinase screening for AMPKα activating kinases. Purified individual kinases from a kinase library were applied to the *in vitro* kinase assays with the bacterial expressed GST-AMPKα1 as substrates. Phosphorylation of the AMPKα1 was detected with the phosphor-specific antibody.

(B-H) The purified kinases from a kinase library are applied to the bacterial expressed GST-AMPKα1 proteins for *in vitro* kinase assays. The phosphorylation of AMPKα is detected by immunoblotting with an antibody targeting the phosphorylated AMPKα Thr172.

(I) A list of kinases that can phosphorylate AMPKα1 based on the *in vitro* kinase screen.

Figure S4. AMPK is activated by RAP2 rather than YAP/TAZ.

(A) Rap2 overexpression regulates phosphorylation of AMPK (pT172) and ACC1 (pACC1). Cells were transfected with Vector or FLAG -Rap2. Phosphorylation of AMPK (pT172) and ACC1 (pACC1) in cells transfected with Vector or FLAG-Rap2A was measured by western blot.

(B) Rap2 regulates AMPK activity. Cells were transfected with Vector or FLAG-Rap2 and cell lysates were taken for AMPK activity assay.

(C) Rap2 deficiency regulates phosphorylation of AMPK (pT172) and ACC1 (pACC1). Rap2 KO cells were cultured on different ECM stiffness. Phosphorylation of AMPK (pT172) and ACC1 (pACC1) in Rap2 KO cells was measured by western blot.

(D) YAP/TAZ regulates phosphorylation of AMPK (pT172) and ACC1 (pACC1). YAP/TAZ KO cells were grown on different ECM stiffness. Phosphorylation of AMPK (pT172) and ACC1 (pACC1) in YAP/TAZ KO cells was detected by western blot.

Figure S5. Correlation of AMPK activity with ECM context.

Matched phosphorylation (ACC_pS79 and AMPKα_pT172) and ECM protein expression (Fibronectin and Collagen VI) data from The Cancer Proteome Atlas were plotted to illustrate the correlation in different cancer types, including 327 Colon adenocarcinoma (COAD) samples from TCGA (A and B), 244 Endometrial Carcinoma samples from MDAAC (C and D), 129 Rectum adenocarcinoma (READ) samples from TCGA (E and F). Pearson's correlation (r) values are indicated within each graph.

Figure S6. Model for coordinated regulation of cell metabolism and cell growth by ECM stiffness.

Under low stiffness, Hippo kinases are activated by RAP2 and phosphorylate both LATS1/2 and AMPKα. The activated LATS1/2 thus phosphorylates YAP/TAZ and inhibits cell growth. In a parallel pathway, the activated AMPK modulates cellular energy metabolism to coordinate proper cell growth.
